# Is a standardised severity index needed in unicoronal craniosynostosis? Challenges in developing an objective metric

**DOI:** 10.1007/s00381-026-07290-0

**Published:** 2026-05-07

**Authors:** Robin van der Straeten, Hanna Lif, Maya Geoffroy, Maxime Taverne, Giovanna Paternoster, Sébastien Laporte, Roman Hossein Khonsari

**Affiliations:** 1 Craniofacial Growth and Form Laboratory, Institut Imagine, Paris, France; 2 Department of Neurosurgery, Vrije Universiteit Brussel (VUB), Universitair Ziekenhuis Brussel (UZ Brussel), Brussels, Belgium; 3 Department of Surgical Sciences, Plastic Surgery, Uppsala University, Uppsala, Sweden; 4 Arts Et Métiers Institute of Technology, Université Paris Nord, IBHGC - Institut de Biomécanique Humaine Georges Charpak, HESAM Université, Paris, France; 5 Department of Neurosurgery, Hôpital Necker – Enfants Malades, Assistance Publique-Hôpitaux de Paris, Paris, France; 6 Department of Maxillofacial Surgery and Plastic Surgery, Hôpital Necker – Enfants Malades, Assistance Publique-Hôpitaux de Paris, Paris, France

**Keywords:** Craniosynostosis, Severity indices, Statistical shape modelling, Principal component analysis

## Abstract

**Purpose:**

Morphological severity of unicoronal synostosis (UCS) is highly variable. Yet, there is no consensus on an objective and standardised metric to quantify 3D severity to evaluate preoperative severity and surgical outcomes. This study aimed to investigate previously described severity indices and compared these to a statistical shape model (SSM) of the cranial vault and expert rating.

**Methods:**

Computed tomography of 77 patients with non-syndromic UCS and 75 healthy controls were included. Principal component analysis (PCA) and partial least squares discriminant analysis (PLS-DA) were used to identify latent axes of shape variation and derived severity estimates, thereby defining an SSM and UCS Skull Shape-based Severity Index (UCS-SI). Expert-based severity ranking served as an independent reference standard. Correlation analyses assessed associations of severity with skull vault shape and expert rankings.

**Results:**

The UCS-SI strongly correlated with expert severity ranking and outperformed classical severity indices. PCA and PLS-DA robustly discriminated UCS from controls, with high variable-importance scores localised to the frontal, supraorbital and parietal regions.

**Conclusion:**

UCS-SI might be a promising severity index in UCS regarding expert perception and objective morphology, offering a clinically interpretable and scalable approach for severity stratification, phenotypic characterisation, and evaluation of surgical outcome trajectories. However, the development of a severity algorithm rather than a severity score might be needed to fully characterise UCS severity. The optimal objective severity metric in UCS is yet to be defined.

## Introduction

Unicoronal synostosis (UCS) is the third most common type of non-syndromic single-suture craniosynostosis [1–4]. It is characterised by fusion of one of the coronal sutures, occasionally associated with additional peri-pterionic suture fusions [5–7]. UCS results in anterior plagiocephaly [1], orbital deformities including asymmetry, Harlequin deformity of the ipsilateral eye [8–11], and scoliosis of the face [8, 12]. Furthermore, these craniofacial features are associated with abnormalities of the skull base [5, 9, 11, 13–22].

UCS present in largely varying severity. Traditionally, shape assessment has relied on cephalometric or subjective observations, making it challenging to quantify and analyse the complex morphological changes occurring during craniofacial growth [13, 14, 23–26]. Recent advancements in computational techniques have led to the emergence of statistical shape modelling (SSM) as a powerful tool for objective and quantitative analysis of craniofacial growth patterns [27].

SSM including principal component analysis (PCA) and partial least squares discriminant analysis (PLS-DA) leverages computational algorithms and statistical methods to analyse the shape and spatial relationships of anatomical structures in craniosynostosis. It provides a data-driven framework that uses geometric data obtained from imaging techniques, such as computed tomography (CT) or 3D photogrammetry, to construct an SSM or 3D morphable model that captures and represents the variations in shape across a population [27, 28]. SSM can be used to assess and quantify skull shape in patients with UCS [5, 29]. Furthermore, SSM enables the establishment of growth trajectories and the prediction of shape development [28, 30, 31].

The aim of the present study was to develop an objective and agnostic way to quantify the overall 3D deformity based on the shape of the external surface of the skull. An SSM model based on PCA and PLS-DA was created. We hypothesise that the score of an individual patient on the latent shape variables quantified how far along the diseases’ primary shape trajectory they fall and thus developed an UCS Skull Shape-based Severity Index (UCS-SI). Finally, this index was evaluated for its correlation with a large set of previously published measures and a novel platform for expert-based ranking.

## Methods

### Data acquisition, preprocessing and registration

Dr. Warehouse [32] at Hôpital Necker-Enfants Malades was used to screen for UCS patients with a preoperative CT-scan of the skull and face between 2006 and 2022. Exclusion criteria included identified pathogenic genetic variants, presence of any syndrome, age > 2 or poor CT quality (movement artefacts, thick slices, large voxel size, not including the whole skull). If multiple preoperative CT-scans were available, only the first one was included. A control cohort of age-matched healthy subjects with CT-scans done for minor trauma or non-deformative pathology was included. A paediatric neurosurgeon and a craniofacial plastic surgeon working clinically with craniosynostosis screened all CT-scans to confirm that all controls were indeed healthy. In the end, 77 UCS patients and 75 healthy controls were included. Characteristics (age, sex, lateralisation) between patients, and between patients and controls, were compared by Wilcoxon signed rank sum tests or Pearson’s Chi-squared tests. The study complied to the Declaration of Helsinki and was ethically approved by the Data Protection Officer of Hôpital Necker–Enfants malades according to previously described principles [33].

Each skull was segmented using 3DSlicer [34] by using the thresholding tool with its default setting at 160 Hounsfield units to only include bone, adapted as necessary for less mineralised skulls. Artefacts and non-cranial bones (mandible, cervical spine) were manually removed. The resulting meshes were decimated to 150,000 polygons. Large holes were manually restored using Meshmixer (Autodesk Inc, 2020, San Francisco, USA). Semi-automatic skull base segmentation was performed in BoneSplit 0.9.2 [35], using a previously described protocol [5]. Orbits were semi-automatically segmented in OrbSeg 0.9.3, as previously described [5, 36]. Skull bases and orbits were mirrored and aligned using Python scripts.

A template skull mesh based on the outer surface of the skull of normal infants was used [31]. After an initial coarse rigid alignment, the template mesh was fitted (wrapped) to align with the segmented CT of each subject using a non-rigid iterative closest point algorithm in the software R3DS guided by a set of anatomical landmarks, as previously described [37]. Hence, a collection of meshes was created where each vertex could be considered being in anatomic correspondence. To negate the effect of laterality in subsequent analyses, all left UCS were mirrored. In summary, cranial surface models underwent dense template-based registration to achieve vertex-wise correspondence, including mirrored instances to balance laterality.

### Statistical shape model

For the purposes of this work, an SSM was constructed by applying PCA to the matrix of Procrustes-aligned 3D coordinate data. PCA decomposes the shape variation into orthogonal principal components (PC), each capturing an independent mode of anatomical variation around the mean shape. Separate SSMs were created for a combined UCS + control cohort (cPC), and a balanced cohort including all mirrored and native instances (bPC). It was assumed the major axes of variance would contain the signature of deformity. As a result, variations in shape not associated with the disease would not be incorporated in the major axes of the model.

On the same coordinate data, a partial least squares discriminant analysis (PLS-DA) was performed, using a binary classifier distinguishing the right-aligned UCS group (R + R′) from controls. For the PLS-DA on the balanced cohort, a dual classifier encoding the presence of pathology (defined as 0/1) and the laterality (defined as −1/+ 1) was used.

**Fig. 1:**
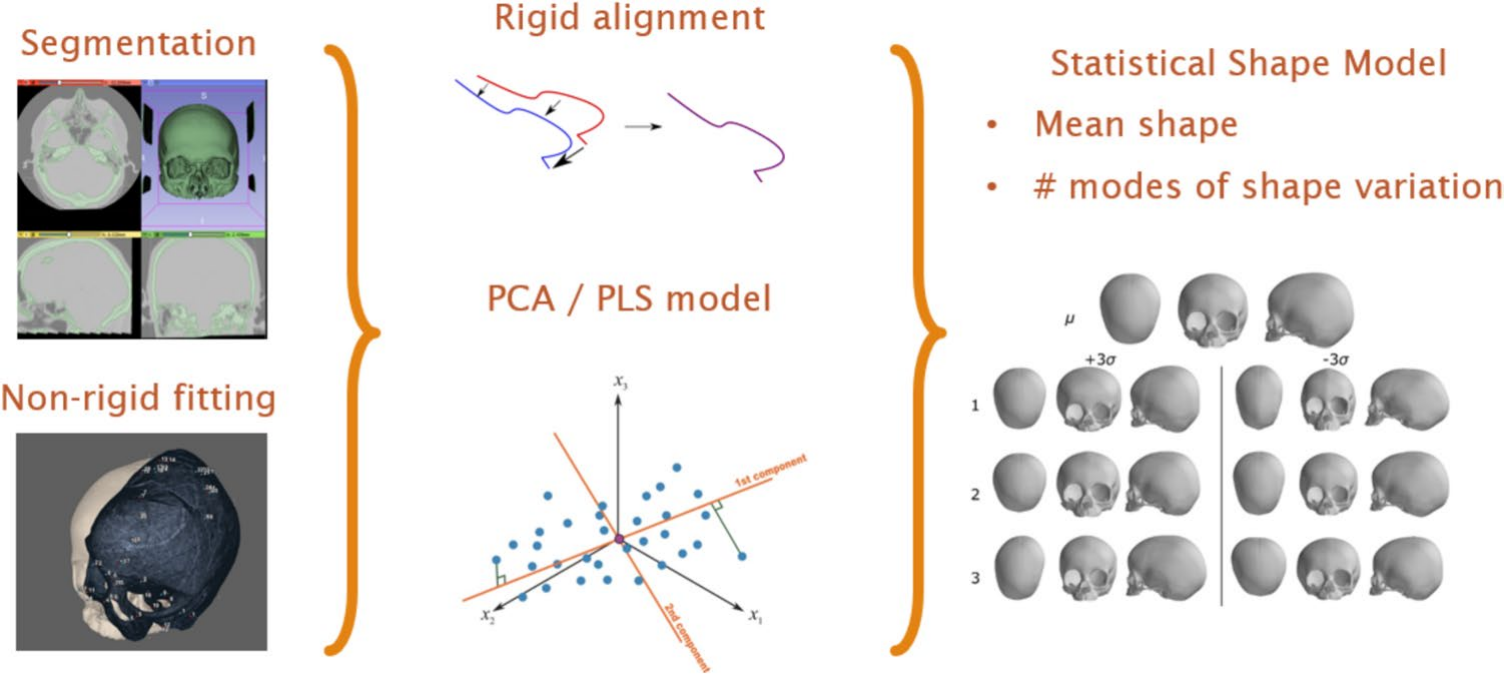
Development of the statistical shape model through the following steps: segmentation, non-rigid fitting, rigid alignment, principal component analysis and partial least squares, calculation of mean shapes and modes of shape variation. PCA, principal component analysis; PLS, partial least squares

PCA can be thought of as naive/unsupervised in the sense that it finds the axes of maximum shape variance. However, these components may not necessarily capture correlation with severity. Even so, it may be the case that the correlation with severity becomes spread across multiple components. In comparison, PLS-DA is supervised and identifies latent variables (LVs) that maximise covariance between shape and diagnostic class. With the aim of optimising the reliability and performance of the SSM model, both PCA and PLS-DA were included. Figure 1 summarises SSM development.

Variable Importance in Projection (VIP) values from the PLS-DA model quantified and visualised how strongly each surface point contributed to discriminating between UCS patients and controls along the latent axis. After fitting PLS-DA, VIP scores summarised the influence of each coordinate on the covariance structure between skull shape and group, integrating information across all retained components. Consequently, high VIP values highlighted regions whose variation was mostly associated with class separation—i.e. areas where shape differences consistently distinguished UCS from controls.

### Cephalometrics, severity indices and expert ranking

In addition to the shape-model–derived metrics, a comprehensive set of severity measures previously proposed in the craniofacial literature was included through a structured PubMed search, to allow for comparison with established indices. The severity measurements included classical cephalometric parameters (anteroposterior length, latero-lateral diameter, cephalic index), cranial vault asymmetry (CVA), the cranial vault asymmetry index (CVAI), hemisphere volume and surface ratios, and the Utrecht Cranial Shape Quantifier (UCSQ) measures (forehead asymmetry ratio, gradient ratios, and peak positions) [26, 38, 39]. We additionally computed vertex-wise asymmetry metrics based on mirrored skulls, as well as area-under-the-curve (AUC) measures derived from the different curves between the left and right sides. In addition, asymmetry scores of the skull base were calculated as the Dice Similarity Coefficient using Python scripts, providing a percentage value between 0 and 100% where 100% represents perfect symmetry [5, 40]. Including validated indices allowed benchmarking of the proposed UCS-SI against existing methods. All statistical testing was performed in R (R Core Team, 2023) with an α-value of 0.05 to determine statistical significance.

To obtain an independent, perception-based reference standard for severity, an expert ranking experiment was conducted on a subset of cases (15 UCS and 2 controls) for which 3D soft tissue segmentations were available. Three craniofacial surgeons performed pairwise comparisons of image pairs and indicated, for each comparison, which case appeared more severe or whether the two were of comparable severity. These pairwise outcomes were modelled using a Bayesian implementation of the Davidson extension of the Bradley–Terry model, which yielded a continuous latent severity score for each subject [41]. Inter-rater reliability was assessed using Kendall’s coefficient of concordance. The resulting rankings were used as the main criterion to assess validity of the statistical shape-based severity index, as well as to benchmark classical cranial asymmetry metrics.

## Results

### Patients and controls

Demographic data is given in Table 1.

**Table 1:** Demographic data

	All, *n* = 152	Controls, *n* = 75	UCS, *n* = 77	*p*-value^a^
**Sex** (median, % of group)				0.01
Female	94 (62%)	39 (52%)	55 (71%)	
Male	58 (38%)	36 (48%)	22 (29%)	
**Age** (months, IQR)	8.0 (5.0, 11.0)	8.0 (4.5, 10.0)	8.0 (5.0, 11.0)	0.3
Right-sided UCS			52 (68%)	
Left-sided UCS			25 (32%)	

UCS = unicoronal synostosis

^a^Pearson’s Chi-squared test; Wilcoxon rank sum test

### Statistical shape model

**Fig. 2:**
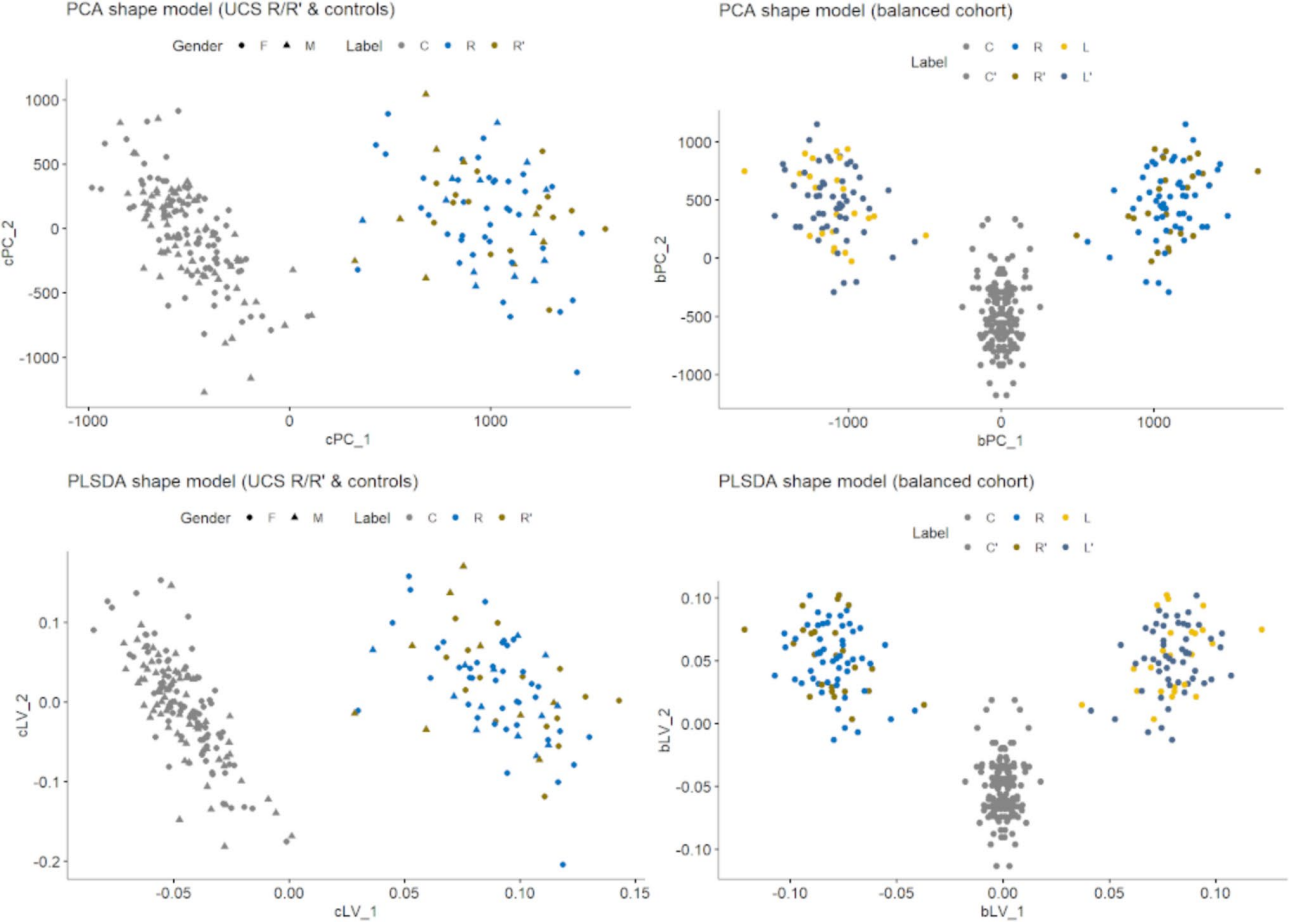
Shape scores (1st and 2nd principal components or latent variables obtained from the statistical shape model. Upper row PCA models. Lower row PLS-DA models. Left: based on combined cohort of right-aligned UCS and controls. Right: balanced cohort with both native and mirrored shape. LV, latent variables; PC, principal component; PLSDA, partial least squares discriminant analysis; UCS, unicoronal synostosis

**Fig. 3:**
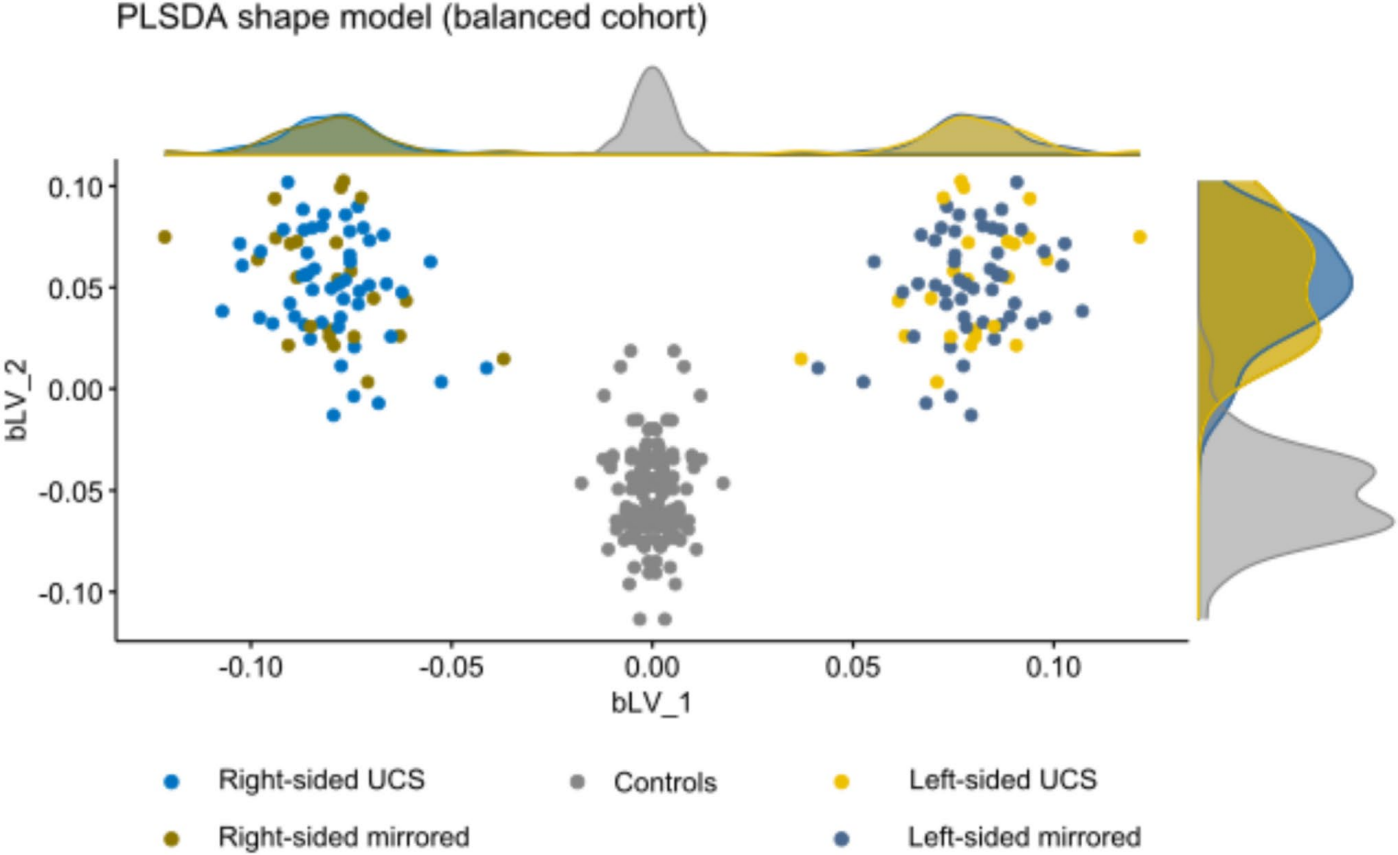
Scoreplot with marginal density plots by group and laterality (1st and 2nd latent variables obtained from the statistical shape model using PLS-DA on the balanced cohort). LV, latent variables; PLSDA, partial least squares discriminant analysis; UCS, unicoronal synostosis

Both for the naive PCA and the supervised PLS-DA models, the first PC/latent variable robustly discriminated UCS from controls (Fig. 2). In the balanced PLS-DA model, the first latent variable (bLV1) showed clear separation, with controls forming a compact cluster centred near zero and UCS cases exhibiting a shifted and more dispersed distribution (Fig. 3). Increasing deviation along bLV1 from the control cluster reflected progressively greater morphological departure from the normal cranial shape, indicating that bLV1 represented the dominant UCS-related deformation axis. The second latent variable (bLV2) provided additional, though weaker, discrimination between UCS cases and controls, with partial overlap between groups. Because variation along bLV2 was invariant to mirroring, this axis could be capturing UCS-related shape differences that are independent of the affected side, suggesting the presence of secondary deformation patterns beyond the primary severity-related axis.

**Fig. 4:**
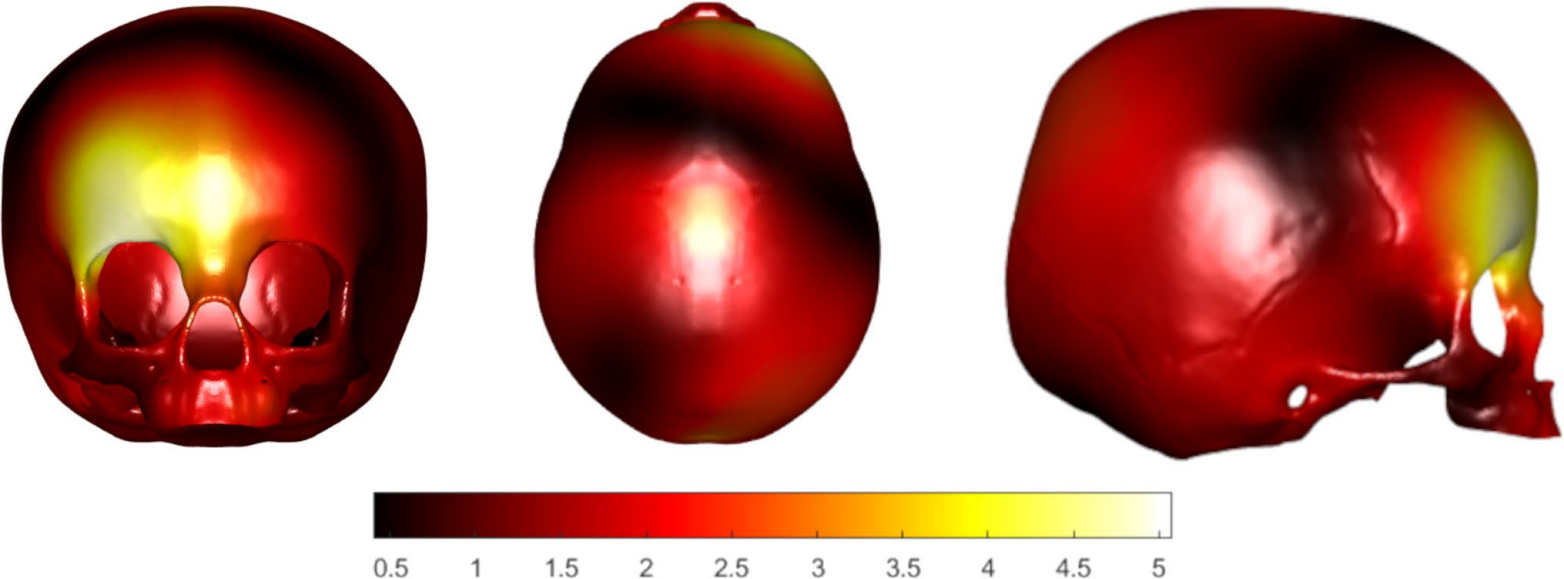
Visualisation of areas specific for unicoronal synostosis compared to controls by calculation of the Variable Importance in Projecting. Warm/intense colours indicate areas that are contributing most to the discrimination between unicoronal synostosis patients and controls compared to darker colours. The right side of the patient represents the synostotic side in patients

VIP scores from the PLS-DA model were mapped onto the skull surface to spatially localise the shape features contributing most to discrimination between UCS patients and controls (Fig. 4). Regions with high VIP values (warm colours, red to yellow) represented areas where shape variation consistently drove class separation, whereas regions with low VIP values (darker colours) contributed little to the discriminative model.

**Fig. 5:**
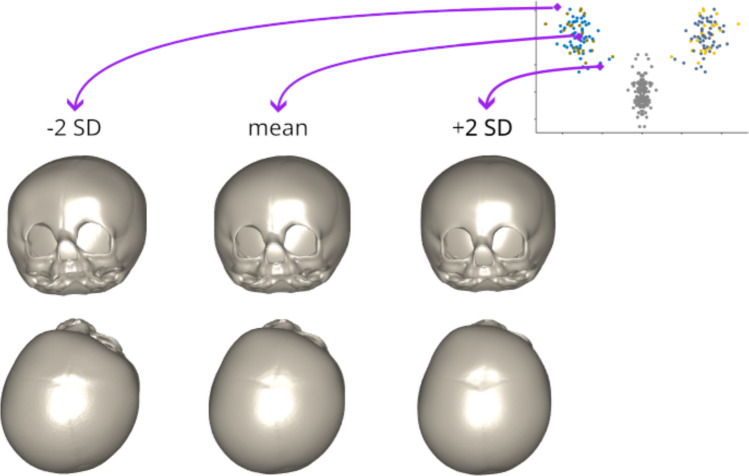
Synthetic skull shapes illustrating severity of unicoronal synostosis along the first two PLS-DA latent variables for the balanced cohort. Shapes were constructed using the mean latent scores of the patient case and ± 2 standard deviations along each latent axis by adding scaled loadings to the mean skull shape. For LV2, the sign of the deviation was inverted to maintain a consistent anatomical progression of severity, reflecting the opposite direction of score change relative to LV1. Increasing UCS-SI values correspond to progressively accentuated frontal and orbital asymmetry

To facilitate anatomical interpretation of the UCS-SI, synthetic skull shapes were generated along the primary PLS-DA latent severity axis. Shapes corresponding to the mean score for UCS cases ± 2 standard deviations were reconstructed by linearly combining the mean shape with the latent variable loadings (Fig. 5). This approach allowed direct visualisation of the progressive morphological changes captured by increasing UCS-SI values.

### Comparison with other indices

**Fig. 6:**
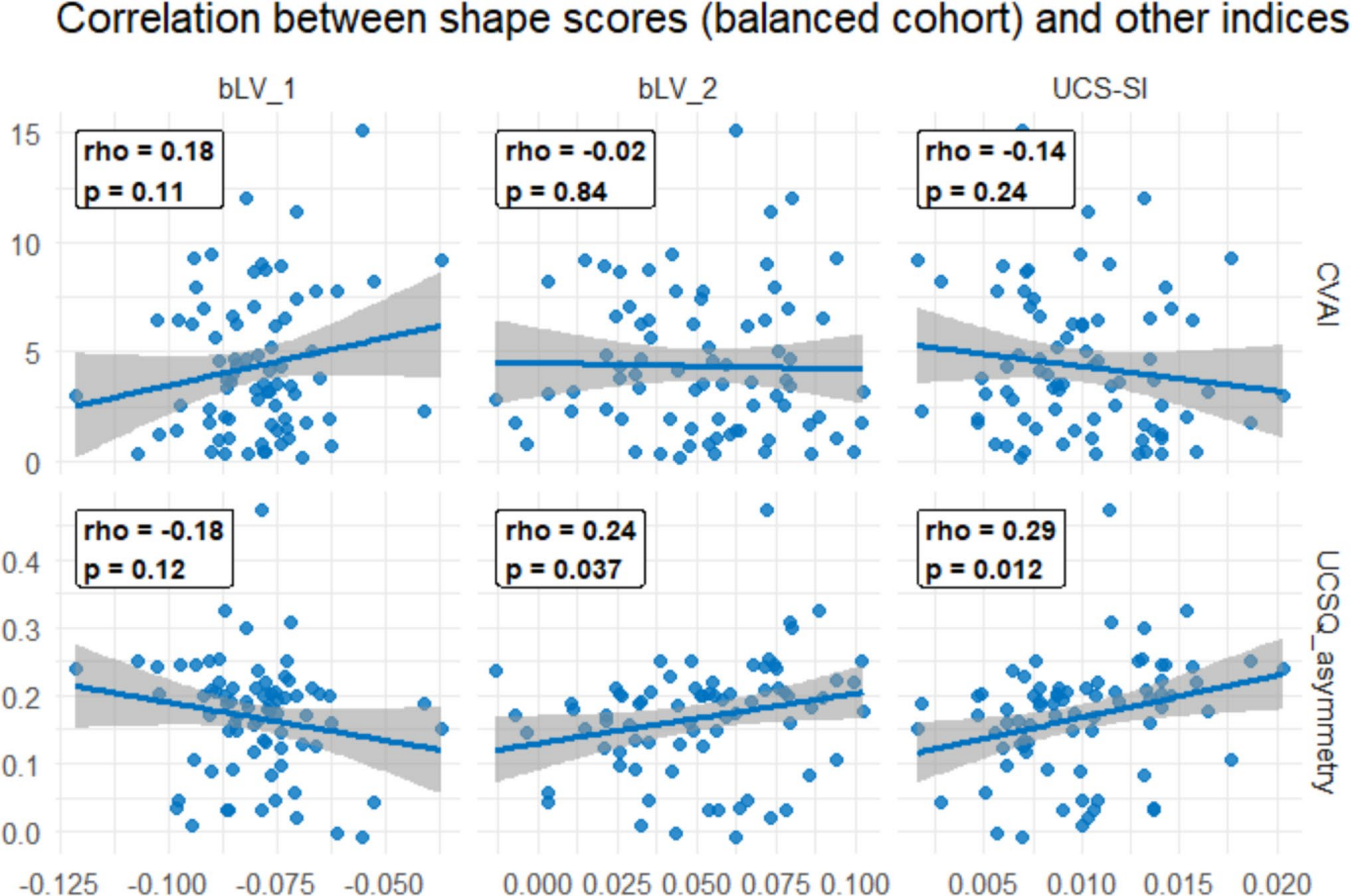
Correlation between shape-based scores (bLV1, bLV2 and UCS-SI) and established UCS severity indices (CVAI and UCSQ). Scatterplots show the Spearman correlation coefficients across the right-sided and mirrored UCS cases (*n* = 76)

Although bLV1 represented the dominant deformation axis, bLV2 contributed additional UCS-related shape variation. Therefore, UCS-SI was defined as a composite measure integrating both latent variables. It was computed as the sum of squares of bLV1 and bLV2, reflecting the overall magnitude of deviation from the control shape distribution in the latent space. Considering only UCS cases, both shape scores showed only weak associations with the established UCS severity indices CVAI and UCSQ. bLV1 demonstrated a somewhat stronger correlation, while bLV2 exhibited additional, independent relationships with UCSQ only. Finally, the composite index UCS-SI was in moderate agreement with UCSQ (Fig. 6).

### Expert ranking

The three raters showed substantial agreement in their pairwise severity judgments (Kendall’s *W* = 0.64, *p* < 0.001). Comparison with morphometric measures revealed strong associations: the expert severity ranking correlated with the primary latent shape axis of the SSM and Dice similarity coefficients. In contrast, traditional cephalometric indices showed weaker or inconsistent correlations (Table 2). Higher absolute Kendall’s τ values indicated closer alignment with expert-perceived severity.

**Table 2:** Correlation between expert ranking and different types of shape and severity measures

	Kendall’s tau	*p*-value
Shape scores		
UCS-SI	− 0.333	0.153
cPC_1	− 0.364	0.116
cPC_2	0.242	0.311
cLV_1	− 0.364	0.116
cLV_2	0.212	0.381
bPC_1	− 0.273	0.250
bPC_2	− 0.333	0.153
bLV_1	0.273	0.250
bLV_2	− 0.394	0.086
Classical cranial vault indices		
Cranial vault asymmetry	0.030	0.947
Cranial vault asymmetry index	0.030	0.947
Utrecht asymmetry index	0.061	0.841
Contour curve difference	0.485	0.031*
Left/right volume proportion		
Hemispheres	− 0.018	1.000
Orbits	− 0.030	0.947
Asymmetry (Dice similarity coefficient)		
Vault	0.345	0.165
Orbits	0.182	0.459
Skull base	0.606	0.005*

*b* balanced cohort including all mirrored and native instances, *c* combined patient + control cohort,* LV* latent variables, *PC* principal component

## Discussion

In this study, a framework for objectively quantifying UCS severity in 3D after evaluation and comparison to previously described methods was presented as the UCS-SI. Unlike CVAI or UCSQ, which quantify predefined geometric asymmetries, UCS-SI captures the dominant axis of UCS-specific shape deformation across the entire cranial vault, aligning more closely with expert holistic assessment. In addition, UCS-SI captures a coherent and anatomically plausible deformation pattern, linking regional discriminative features to a continuous, clinically interpretable severity trajectory. This severity index integrated expert perception and objective morphology, offering a clinically interpretable and scalable approach for severity stratification, phenotypic characterisation, and evaluation of surgical outcome trajectories. However, further studies are needed to evaluate its clinical relevance. Developing a severity algorithm rather than a severity score might be needed to fully characterise UCS severity.

### Clinical implications

The purpose of developing a stable severity index and unifying the field around one single method for quantifying UCS severity would allow for comparison between surgeons, surgical treatment methods and craniofacial centres. Consequently, research derived from a unified index would improve our understanding of UCS severity and complexity, allow for studying the poorly understood relationship between shape and function, and potentially improve treatment through constructing the foundation to large multi-centre studies based on long-term evaluation of severity over time. In addition, it would enable improved prognosis and thereby better-informed patients and parents. While the present implementation requires specialised software and preprocessing, the computation of UCS-SI itself is fully automated once the shape model is established, making routine clinical use feasible in centralised settings. As a long-term purpose, the development of a stable severity index could allow for individualised surgical treatment based on preoperative characteristics. Following these arguments, this study implies that an objective and globally used UCS severity metric is indeed needed to improve the care of UCS patients.

### Developing an objective UCS severity metric

A UCS severity metric serving a stable purpose in both research and clinical practice needs to be simple enough for clinical use but complex enough to provide sufficient sensitivity and specificity to capture the full range of complexity in UCS morphology. We submit that an ideal severity metric in UCS should satisfy the following requirements:

- Being a robust objective measurement or a combination of multiple measurements.
- Correlate with UCS-specific changes in morphology (asymmetry).
- For comparability, a specific value (e.g. “0”) should correspond to a theoretical perfectly symmetric skull.
- Preferably, a definable threshold would allow for clear discrimination between UCS and controls. Above this threshold, an increasing (absolute) value should correspond to a more severe UCS phenotype.
- A symmetric metric for left and right-sided UCS; either the index is the same regardless of lateralisation, or the sign of the index indicates the ipsilateral side.
- The metric is invariant with age or can be corrected for age in a straightforward manner.
- The deformational nature of UCS makes it difficult to define a midline, any asymmetry index must find a stable way to define a midline or be based on methods that do not depend on this.
- To strengthen objective evaluation, UCS severity should preferably be defined as the degree of dissimilarity to controls rather than similarity to expert rating. Expert rating is however needed to develop a clinically relevant index.

This study does not represent the first effort to unify the field on one method to quantify severity in non-syndromic UCS [3, 27]. Undoubtedly, it has been a challenge to reach agreement on a single methodology to serve this purpose. In metopic craniosynostosis, larger success has been reached in this regard [42]. There is an ongoing discussion supported by rigorous research on the indications for operating patients with metopic craniosynostosis. Thereby a severity index provides a clear aim, benefit and end goal of unifying the field on one method to quantify severity in metopic craniosynostosis. In UCS, there is not one clear superior surgical treatment method although less invasive unilateral methods such as fronto-orbital distraction osteogenesis and endoscopic strip craniectomy with helmet have proven superior to fronto-orbital advancement in a number of limited cohort studies [43–46]. However, the evidence proving that one method is superior regardless of age, severity, surgeon-specific factors and small or large-centre setting is lacking. There are currently no published studies that have followed UCS patients treated with different surgical treatment methods until the end of craniofacial growth. Given the large variability in UCS severity, one treatment approach might not be beneficial for all UCS patients, thereby stressing the relevance for developing a severity metric to enable individualised treatment based on preoperative characteristics. Even though one severity index might be enough for metopic craniosynostosis, this study suggests that a single value might not completely capture the complex phenotypical UCS variation. Consequently, there might be indications favouring developing an algorithm of UCS severity rather than a single severity index. Combining SSM with artificial intelligence could be the next logical step in this regard. If successful, this could lay the foundation for developing complex severity algorithms in other types of craniofacial malformations including syndromic craniosynostosis in the future.

In summary, SSM including UCS-SI offers a powerful and data-driven approach to quantify morphological UCS severity. It provides a comprehensive understanding of associated UCS shape changes, possibly enabling improved treatment planning and outcome prediction in the future. By providing a quantitative and objective approach to craniofacial severity and growth, this tool holds great promise in advancing our knowledge of craniofacial development with the potential to improve the care of UCS patients.

### Limitations

The sample size would have been larger if multiple centres were included; only including data from one centre however limited the variability of examination protocols and CT scanner settings. Only low-dose scans of high quality were included, limiting the sample size further. Few patients were included in the expert ranking. 3D photogrammetry instead of CT might have been preferred for expert ranking. The study had a cross-sectional design, thereby investigating independent subjects compared to a longitudinal design. It was not possible to perform repeated CT scans of the same subjects due to the cautious approach of radiation exposure. Postoperative outcomes were not analysed, as longitudinal CT imaging was not available for this cohort. Consequently, the relationship between preoperative UCS-SI and surgical outcome remains to be established and represents a critical next step for validation.

UCS severity might vary for multiple reasons that were not investigated fully, such as age, sex, genetics, environmental-, biomechanical- and biochemical factors. A template of 150,000 vertices on the outer surface of the skull allowed for holistic evaluation of the vault shape in comparison to more limited landmarking and semi-landmarking techniques; the law of large number should however be considered. The data points in this study were classic 3D Cartesian coordinates of a dense representation of the outer skull surface. Other embeddings could also be considered, including left–right symmetry separating if corresponding points move in synergy or opposed; or an embedding using the deviation from a normal/reference shape. Only the skull vault was included; future studies should preferably also include the skull base and face, preferably by combining CT with 3D photogrammetry.

### Conclusion

UCS-SI constitutes a promising severity index in UCS regarding expert perception and objective morphology, offering a clinically interpretable and scalable approach for severity stratification, phenotypic characterisation, and evaluation of surgical outcome trajectories. However, the development of a severity algorithm rather than a severity score might be needed to fully characterise UCS severity. The optimal objective severity metric in UCS is yet to be defined.

## Data Availability

Scripts, analyses and anonymised data sets are available upon request to the corresponding author.
